# *Saccharomyces cerevisiae* biofilm tolerance towards systemic antifungals depends on growth phase

**DOI:** 10.1186/s12866-014-0305-4

**Published:** 2014-12-04

**Authors:** Rasmus Bojsen, Birgitte Regenberg, Anders Folkesson

**Affiliations:** Department of Systems Biology, Technical University of Denmark, Kgs. Lyngby, Denmark; National Veterinary Institute, Technical University of Denmark, Frederiksberg, Denmark; Department of Biology, University of Copenhagen, Copenhagen, Denmark

**Keywords:** Yeast, Biofilm, Drug tolerance, Antifungal agent, Amphotericin B, Voriconazole, Flucytosine, Caspofungin, Fungicide, Resistance

## Abstract

**Background:**

Biofilm-forming *Candida* species cause infections that can be difficult to eradicate, possibly because of antifungal drug tolerance mechanisms specific to biofilms. In spite of decades of research, the connection between biofilm and drug tolerance is not fully understood.

**Results:**

We used *Saccharomyces cerevisiae* as a model for drug susceptibility of yeast biofilms. Confocal laser scanning microscopy showed that *S. cerevisiae* and *C. glabrata* form similarly structured biofilms and that the viable cell numbers were significantly reduced by treatment of mature biofilms with amphotericin B but not voriconazole, flucytosine, or caspofungin. We showed that metabolic activity in yeast biofilm cells decreased with time, as visualized by FUN-1 staining, and mature, 48-hour biofilms contained cells with slow metabolism and limited growth. Time-kill studies showed that in exponentially growing planktonic cells, voriconazole had limited antifungal activity, flucytosine was fungistatic, caspofungin and amphotericin B were fungicidal. In growth-arrested cells, only amphotericin B had antifungal activity. Confocal microscopy and colony count viability assays revealed that the response of growing biofilms to antifungal drugs was similar to the response of exponentially growing planktonic cells. The response in mature biofilm was similar to that of non-growing planktonic cells. These results confirmed the importance of growth phase on drug efficacy.

**Conclusions:**

We showed that *in vitro* susceptibility to antifungal drugs was independent of biofilm or planktonic growth mode. Instead, drug tolerance was a consequence of growth arrest achievable by both planktonic and biofilm populations. Our results suggest that efficient strategies for treatment of yeast biofilm might be developed by targeting of non-dividing cells.

## Background

Nosocomial fungal infections are a major problem for immune compromised patients with a severe underlying disease [1]. Fungi can cause infections by colonizing mucosal surfaces in the oral cavity, airways, wounds and the gastrointestinal tract [2]. Fungi can also adhere to invasive medical devices and cause severe septicemia upon detachment [3]. The hallmarks of biofilms are surface attachment and production of an extracellular matrix (ECM) [4]. Failure to eradicate microbial infections is often attributed to the unique lifestyle of cells in biofilms and it is widely accepted that cells in a biofilm possess antimicrobial tolerance mechanisms that are distinct from their planktonic counterparts [2].

Drugs currently being used to treat systemic mycoses belong to four major classes. The azoles target cytochrome P450 and inhibit cell membrane ergosterol biosynthesis, resulting in accumulation of toxic ergosterol intermediates [5]. Azoles have poor efficacy against *Candida* species other than *C. albicans*, such as *C. glabrata* [6]. The number of nosocomial blood isolates of these non-susceptible *Candida* species has increased in the past decades, possibly because of the selection that frequent azole use impose [7]. The echinocandins inhibit 1,3-β-glucan synthases, resulting in a reduction in cell wall 1,3-β-glucan [8], and the polyenes target ergosterol and cause pore formation in the fungal cell membrane [9]. The fourth class is the antimetabolite flucytosine. Flucytosine is deaminated upon uptake in susceptible cells and converted to 5-fluorouridine triphosphate, which is incorporated into RNA, inhibiting protein synthesis [10]. Flucytosine can also be converted to 5-fluorodeoxyuridine monophosphate which acts on thymidylate synthase to inhibit DNA synthesis [10]. Despite the pronounced diversity in antifungal mechanism of action and chemical structure, most antifungal agents are inactive against fungal biofilms [11].

Several mechanisms have been suggested to be responsible for drug tolerance of yeast biofilms. One of them is the ECM layer that contains β-1,3 glucans and extracellular DNA [12,13]. Treatment of biofilm cells with glucanases or DNase result in increased efficacy of antifungal agents, which indicate a role of ECM on antifungal drug tolerance [13,14]. However, it has been shown that antifungal susceptibility is independent of amount of matrix produced and antifungal drugs can diffuse through the matrix layer in inhibitory concentrations [15,16]. The ECM, in combination with the nutrient-limited environment that results from a large number of microbial cells, might induce expression of genes that help cells cope with stressful conditions. Altered gene expression could involve differential regulation of general stress-response genes that affect drug tolerance. For example, efflux pumps are reported to be upregulated in young and intermediate [17,18] biofilms in *Candida* species. However, efflux pump knockout mutants remain drug resistant [18,19] and up-regulation is lost in mature biofilms [17,18]. Furthermore, since polyenes and echinocandins are not a substrate of any known efflux pumps [20], efflux pumps are not responsible for biofilm-mediated tolerance to these drug classes. None of the suggested tolerance mechanisms are solely responsible for the multidrug tolerance associated with biofilm, and it might be a combination of several individual mechanisms that cause multidrug tolerance in yeast biofilms.

*Candida* is the most frequent cause of fungal infections and extensive research has been performed with this organism to investigate regulation of biofilm formation and antifungal drug recalcitrance [3]. However, due to a limited repertoire of genetic and molecular techniques available for some *Candida* species, the knowledge about yeast biofilm regulation and drug tolerance is incomplete. The genetic tractability of another fungus, *Saccharomyces cerevisiae*, has made it a model organism for the study of fundamental issues in fungal biology [21]. Transition from yeast to filamentous morphology is correlated to virulence in *Candida albicans* and key signaling pathways controlling this process is conserved in *S. cerevisiae* [22]. *Candida glabrata* is phylogenetically more closely related to *S. cerevisiae* than to other *Candida* species [23] and they have homologous cell-surface adhesins [24]. *C. glabrata* and *S. cerevisiae* furthermore form biofilms as haploids with similar biofilm architecture: thin layer of biofilm cells with yeast morphotype surrounded by a low density of ECM [25,26]. *S. cerevisiae* is therefore relevant for the study of *C. albicans* virulence and *C. glabrata* biofilm. *S. cerevisiae* has previously been used as a model organism to study yeast biofilm development and regulation by taking advantage of the molecular tools available for this organism [27-33]. However, much less effort has been made to investigate the response of *S. cerevisiae* biofilm cells to antifungal treatment [32,34,35]. *S. cerevisiae* has the potential to cause human infections [36] and its ability to adhere to plastic surfaces [28,30] makes it a relevant organism for the study of yeast biofilm tolerance towards antifungal agents.

Fungal and bacterial research report 1000-fold higher tolerance level of mature biofilms compared to proliferating planktonic populations [37,38]. Research in bacteria has shown that the tolerance phenotype is similar between biofilm and planktonic cells when cultivated for equally long time in identical medium [39-41]. This indicates that tolerance mechanisms are not biofilm-specific and that planktonic cells can achieve the same level of tolerance. To address if growth arrest is also relevant for drug tolerance in yeast biofilm, we have compared susceptibility of common antifungals in biofilms and planktonic cells cultivated under similar conditions. We used *in vitro* biofilms of *S. cerevisiae* and *C. glabrata* cultures to investigate antifungal tolerance to drugs from each of the major antifungal drug classes used for systemic treatment of human pathogenic fungal infections: the polyene amphotericin B (AmB), the azole voriconazole (VOR), the antimetabolite flucytosine (5FC), and the echinocandin caspofungin (CAS). We found that the ability of biofilms to survive antifungal treatment was dependent on the mode of action of the antifungal agent and the growth state of the yeast cells.

## Results

### *S. cerevisiae* and *C. glabrata* biofilms have similar structure and antifungal tolerance

We initially determined antifungal drug susceptibilities of exponentially growing planktonic *S. cerevisiae* and *C. glabrata* cells towards four antifungal compounds, AmB, VOR, 5FC, and CAS. Drug susceptibilities of *S. cerevisiae* cells were similar to *C. glabrata* as determined by minimal inhibitory concentrations (MIC), except for VOR that was 4 μg/ml against *C. glabrata*, and 1 μg/ml against *S. cerevisiae* (Table 1).

**Table 1:** **MIC susceptibility pattern of antifungal agents against** ***S. cerevisiae*** **and** ***C. glabrata***

**Organism**	**MIC** **(** **μg/** **ml)**			
	**VOR**	**5FC**	**CAS**	**AmB**
***S. cerevisiae***	1	8	1	1
***C. glabrata***	4	8	0.5	2

*VOR*: voriconazole, 5*FC*: flucytosine, *CAS*: caspofungin, *AmB*: amphotericin B.

**Figure 1:**
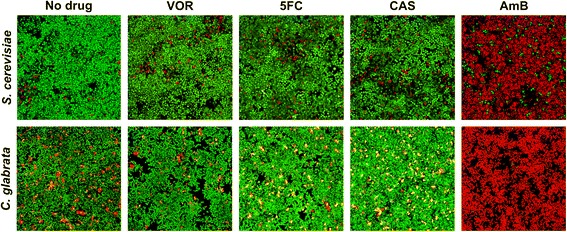
**Mature** ***S. cerevisiae*** **and** ***C. glabrata*** **biofilms have similar sensitivity to antifungal drugs.** Antifungal drug activity against 48-hour biofilms was visualized by confocal laser scanning microscopy. GFP-tagged *S. cerevisiae* was stained with propidium iodide (PI, 1 μM) to mark living (green) and dead (red) cells. *C. glabrata* was stained with Syto9 (3 μM) and PI (1 μM) to mark living (green) and dead (red/yellow) cells. Biofilm cells were treated for 24 hours with the indicated antifungal agents. VOR: voriconazole (10 μg/ml for *S. cerevisiae* and 50 μg/ml for *C. glabrata*), 5FC: flucytosine (80 μg/ml), CAS: caspofungin (10 μg/ml for *S. cerevisiae* and 5 μg/ml for *C. glabrata*), and AmB: amphotericin B (10 μg/ml for *S. cerevisiae* and 20 μg/ml for *C. glabrata*).

Yeast biofilm architecture and antifungal drug sensitivity was investigated using confocal laser scanning microscopy (CLSM). Mature GFP-tagged biofilm cells were challenged with an antifungal agent for 24 hours and stained with propidium iodide (PI) to identify dead cells. *S. cerevisiae* biofilms contained a thin layer of cells (approximately 30 μm) with a few dead cells distributed throughout the biofilm. Biofilms treated with VOR, 5FC, or CAS had the same architecture and mixture of living and dead cells as untreated control cells (Figure 1), showing that the drugs were inactive against yeast biofilms. AmB was the only tested drug with anti-biofilm activity, killing most cells after 24 hours (Figure 1). The small subpopulation of cells that survived AmB treatment was randomly distributed in the biofilms.

To determine if results from the *S. cerevisiae* biofilm model applied to drug susceptibility in a pathogenic yeast, we investigated the antifungal drug susceptibility of *C. glabrata* biofilms. *C. glabrata* was cultivated under conditions similar to *S. cerevisiae* cultures and developed a thin layer of biofilm cells (approximately 25 μm). After 48 hours, mature biofilms were challenged with an antifungal drug for 24 hours and stained with Syto9 and PI to visualize living and dead cells. Results were similar to those obtained with *S. cerevisiae*. Most *C. glabrata* biofilm cells exposed to VOR, 5FC or CAS showed living cells with a few dead cells distributed in the biofilm, similar to the appearance of the untreated control cells (Figure 1). AmB treatment killed most cells with a small, surviving subpopulation randomly distributed in the biofilm. These results suggested that *S. cerevisiae* could be used as a model organism to study antifungal tolerance in biofilms of the pathogenic *C. glabrata*.

### Metabolic activity of biofilm cells decreases with biofilm maturity

**Figure 2:**
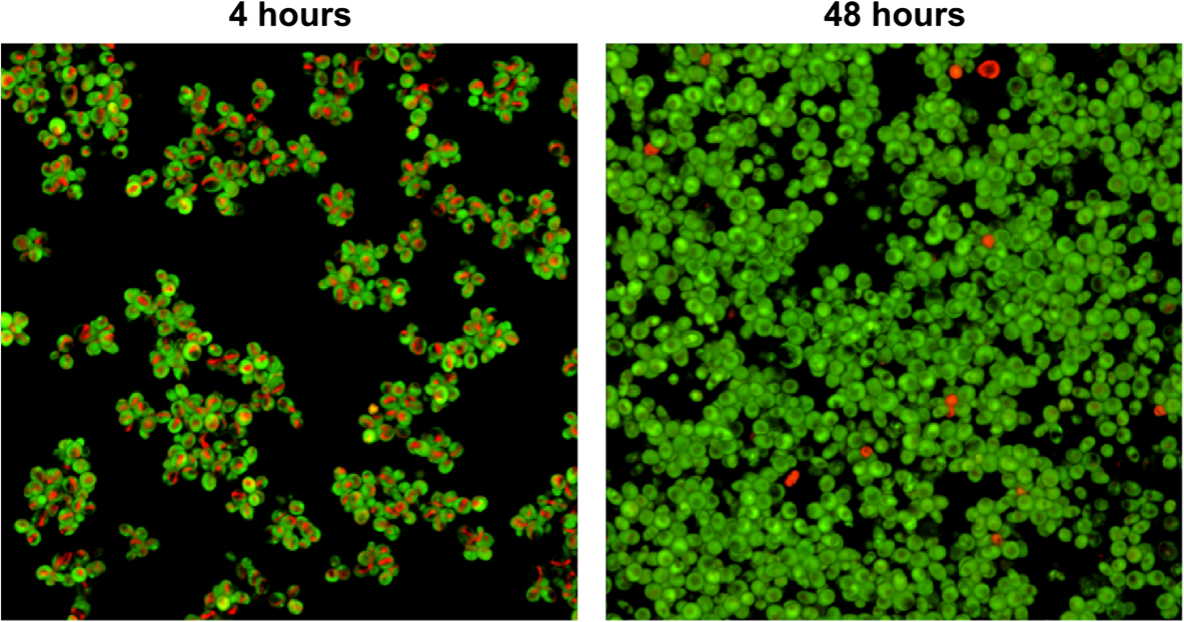
**Metabolic activity of yeast cells in biofilms decreases with incubation time.** *S. cerevisiae* cells stained with FUN-1 (10 μM) after incubation of biofilm for 4 hours and 48 hours respectively. Metabolically active cells produce red cylindrical intravacuolar structures.

Planktonic microbial cells cultivated in a closed system take up nutrients from the environment and enters a stationary growth state with decreased metabolic activity when nutrients become limited. To investigate if the metabolic activity of biofilms at 48 hours was reduced compared to a 4 hour biofilm, we measured metabolic activity using FUN-1 staining. FUN-1 permeabilizes the plasma membrane and biochemical processing of the dye by an unknown pathway identifies metabolically active cells with intravacuolar structures [42]. Most cells in a 4-hour biofilm showed high metabolic activity as estimated by staining intensity of the vacuole (red color, Figure 2), but staining decreased with biofilm incubation time. After 48 hours, only a small subpopulation of cells in the biofilms showed FUN-1 staining of vacuoles indicating a lower or different metabolic activity in mature biofilm than that found in young 4 hour biofilm (Figure 2).

### Activity of antifungal drugs depends on growth state

**Figure 3:**
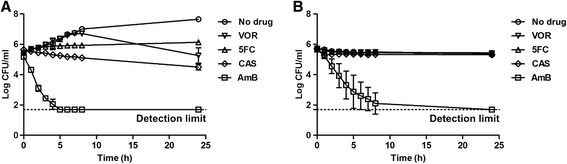
**Antifungal drug susceptibility in exponentially growing and growth arrested planktonic cells.** **(A)** Killing kinetics of exponentially growing planktonic *S. cerevisiae* cultivated in 2% glucose exposed to 5 times the MIC of the indicated antifungal drugs. **(B)** Killing kinetics of growth-arrested planktonic *S. cerevisiae* cultivated in carbon-depleted minimal medium exposed to 5 times MIC of the indicated antifungal drugs. VOR: voriconazole (5 μg/ml), 5FC: flucytosine (40 μg/ml), CAS: caspofungin (5 μg/ml), AmB: amphotericin B (5 μg/ml). n = 3, error bars show standard deviations.

We hypothesized that the limited metabolic activity and resulting lack of growth observed in cells in mature biofilms may be an important cause of the low antifungal activity of the drugs tested. We measured therefore the killing kinetics of the antifungals using an exponentially growing planktonic population and a growth-arrested planktonic population. Untreated control cells proliferated with a doubling time of 1.5 hours in the exponential growth phase for the first 8 hours of incubation (Figure 3A). The density of cells exposed to VOR increased at the same rate as the untreated sample for the first 7 hours of incubation. Subsequently, the azole drug inhibited growth, resulting in a decrease in viability after 24 hours. After two hours of exposure to 5FC, the growth of exponential phase populations was inhibited and cells remained at the same viability and density for 24 hours showing that 5FC had fungistatic activity. CAS had an inhibitory effect on exponential growth within the first hours of exposure and a consistent killing rate throughout the experiment that resulted in a 10-fold reduction in colony forming units (CFUs) after 24 hours compared to the initial population. Challenging cultures with AmB rapidly decreased the viable population, reaching the lower detection limit for CFUs after 5 hours.

We next investigated how growth arrest affects susceptibility to antifungal agents by incubating cells in carbon-depleted medium. Starting cell density was similar to the starting density used for time-kill studies on the exponential growing populations to eliminate cell numbers from affecting comparisons between the two experiments. Growth-arrested *S. cerevisiae* exposed to VOR, 5FC or CAS had viability similar to untreated control cells, showing that the drugs had no activity against non-growing cells in stationary phase (Figure 3B). Cells exposed to AmB were killed, but the killing rate was lower than the rate observed for exponentially growing cells. The lower detection limit for CFUs was not reached in the first 8 hours of drug exposure, but only after 24 hours.

### Drug sensitivity restored in a growing biofilm population

**Figure 4:**
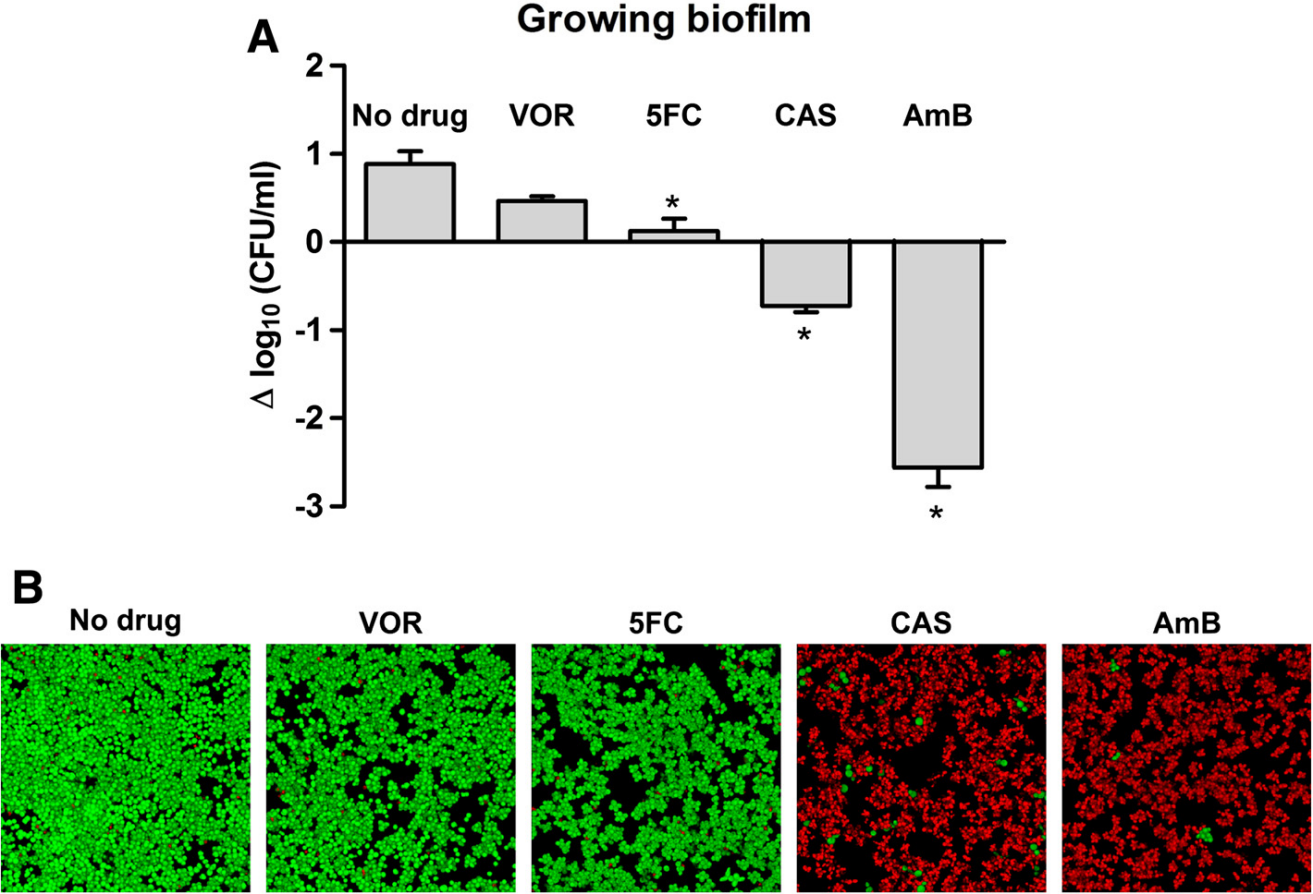
**Antifungal drugs are active against growing biofilms.** **(A)** Quantitation of biofilm cell viability. Biofilms were grown for 4 hours and viability determined as CFUs. Biofilms were then treated with 10 times the MIC of the indicated antifungal drug for 24 hours and CFUs were measured. Shown is log change in CFUs after 24 hours treatment. n = 3, error bars are standard deviations. Statistical significance between treated and untreated samples was evaluated with Student's t-test. *P < 0.01. **(B)** GFP-tagged *S. cerevisiae* in 4-hour biofilms treated for 24 hours with the indicated antifungal agents. VOR: voriconazole (10 μg/ml), 5FC: flucytosine (80 μg/ml), CAS: caspofungin (10 μg/ml), and AmB: amphotericin B (10 μg/ml). Cells were visualized as described in the legend to Figure 1.

The antifungal activity of 5FC, CAS and AmB against exponentially growing planktonic *S. cerevisiae*, but not against growth-arrested cells, suggested that drug activity depended on cell growth. To test if growth-dependent drug activity also applied to cells in biofilms, the viability of a growing *S. cerevisiae* biofilm was quantified as CFUs and visualized with CLSM. Quantification and visualization assays were conducted after 24 hours of drug treatment. The number of cells in untreated 4-hour biofilms increased 8-fold over 24 hours, as determined by CFUs (Figure 4). Growth in 4-hour biofilms exposed to VOR or 5FC was inhibited compared to untreated control biofilms. Cell numbers determined by CFUs increased 3-fold with VOR treatment and 1.3-fold with 5FC treatment. In contrast, 80% of biofilm cells exposed to CAS were killed, with surviving cells sporadically distributed in the biofilm (Figure 4). AmB had a fungicidal effect on young biofilms, killing 99.7% of cells during a 24-hour exposure. Biofilms treated with AmB still contained minor surviving subpopulations (Figure 4).

### Mature biofilm and stationary planktonic yeast have similar susceptibility to systemic antifungals

We observed that most drug classes tested in this study were inactive against mature biofilms (Figure 1) and planktonic cells that are arrested for growth (Figure 3), whereas growing biofilm and planktonic cells were susceptible to both AmB, VOR, 5FC, and CAS. These data suggest that the physiological state of the cell in response to ceased proliferation, rather than a biofilm-specific response mediate drug tolerance in yeast biofilms.

To determine the effect of growth arrest on drug tolerance, we investigated differences in antifungal drug susceptibility between stationary cultures of cells grown planktonically or in biofilms for 48 hours. *S. cerevisiae* biofilms were cultivated on flat polystyrene surfaces. For planktonic control populations, *S. cerevisiae* was cultivated in glass tubes [43]. An isogenic biofilm-deficient *flo11* knockout mutant was included as a negative control for biofilm formation [28]. The average inoculum before drug challenge was 1.6 × 10^7^ CFU/ml for biofilm cells, 1.8 × 10^7^ for planktonic cells and 7.5 × 10^6^ for *flo11*, minimizing the influence of different cell densities on drug susceptibility between the cultivation assays.

**Figure 5:**
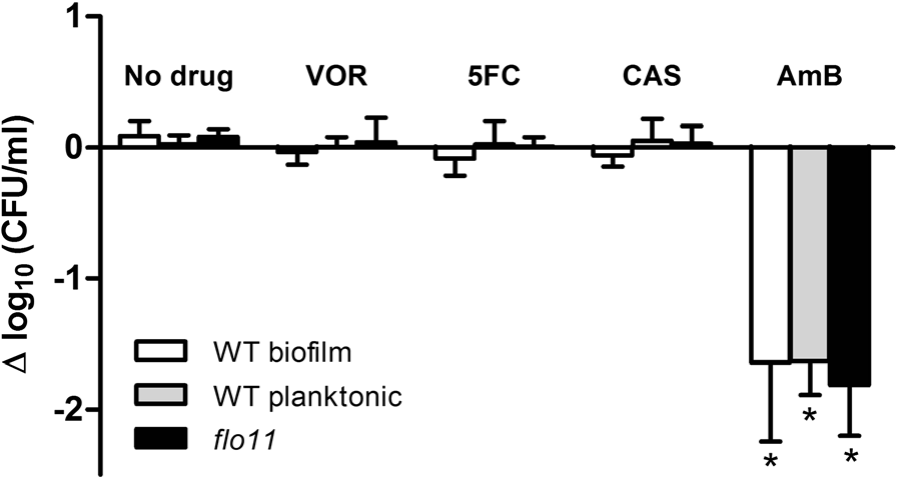
**Stationary** ***S. cerevisiae*** **cells grown in biofilms and planktonic cultures have similar drug tolerance.** Cultures were grown for 48 hours and viability was measured as CFUs. Cells were treated with 10 times MIC of the indicated antifungal drug, or left untreated as a control, and CFUs were measured after 24 hours. Shown is log change in viability. White bar: wild type *S. cerevisiae* Σ1278b grown as biofilms on polystyrene surfaces. Grey bar: wild type grown planktonically in glass flasks. Black bar: *flo11* knockout mutant grown planktonically in glass flasks. VOR: voriconazole (10 μg/ml), 5FC: flucytosine (80 μg/ml), CAS: caspofungin (10 μg/ml), AmB: amphotericin B (10 μg/ml). n = 3, error bars show standard deviation. Statistical significance between treated and untreated samples was evaluated with Student’s t-test. *P < 0.01.

All three cultures, biofilm, *flo11* control, and planktonic, were challenged for 24 hours with antifungal agents. No significant effects on CFUs were seen after treatment with VOR, 5FC, or CAS (Figure 5), indicating that growth arrested *S. cerevisiae* was not susceptible to any of the drugs under any of the three growth conditions. Only treatment with AmB significantly decreased population sizes (P < 0.01, Student’s *t*-test). Exposure to AmB killed 95-98% of the yeast populations regardless of growth condition (Figure 5). However, in all three growth conditions, a subpopulation of 2-5% of cells survived drug treatment, so AmB was unable to eradicate the entire *S. cerevisiae* population.

## Discussion

In the current study, we found that antifungal drug efficacy against *S. cerevisiae* biofilm was dependent on cell growth. Only growing *S. cerevisiae* cells were susceptible to growth inhibition by the fungistatic drugs VOR and 5FC, and killing by CAS. However, *S. cerevisiae* cells in both growing and stationary state were efficiently killed by AmB. We further observed that the effects of antifungals were independent of biofilm or planktonic modes of growth.

Multidrug tolerance mechanisms in biofilms are suggested to include production of an ECM and a densely packed microbial structure that shields cells, preventing antimicrobials from reaching their targets. Cell-surface proteins in the Flo family are responsible for *S. cerevisiae* adhesion and ECM production [44]. Flo11p is the only flocculation protein expressed in the Σ1278b *S. cerevisiae* strain and it is essential for biofilm formation in liquid medium [28,44-46]. We showed that a *flo11* mutant has an antifungal tolerance phenotype that is similar to mature yeast biofilms (Figure 5). This finding suggests that cell-cell adhesion and Flo11p-dependent matrix production are not obstacles for cell penetration by antifungal drugs in *S. cerevisiae* biofilm. This observation is in contrast to the drug sequestering role of matrix β-1,3 glucan in *Candida* biofilms [13,47,48] and might reflect differences in matrix composition between Σ1278b *S. cerevisiae* and *Candida* biofilms. The ECM layer is, however, not the sole contributor to the drug tolerant phenotype because systemic antifungals can penetrate the ECM of *Candida* species biofilms at concentrations that exceed the MIC values, and no correlation is observed between the amount of matrix produced and drug susceptibility [15,49]. In agreement with this, our results show similar drug susceptibility between *S. cerevisiae* and *C. glabrata* biofilms, suggesting another important contributor to the observed antifungal drug tolerance phenotype.

Heterogeneous microbial biofilms often contain large subpopulations with low metabolic activity [50,51]. We showed that the metabolic activity of most cells in yeast biofilms decreased as the biofilm matured (Figure 2) and we observed no increase in cell density in mature biofilms (Figure 5A). Therefore, large fractions of cells in mature biofilms are likely in a stationary state. Even though antimicrobial agents have diverse modes of action, most are dependent on active growth to kill cells [52], which we confirmed in the present study.

The drug 5FC has fungistatic activity against *Candida* species [53]. We found a similar fungistatic activity against proliferating *S. cerevisiae* Σ1278b planktonic and biofilm populations (Figure 3A and Figure 4). However, 5FC was inactive against mature biofilms and growth arrested planktonic cells (Figures 1, 3B and 5). This result is not surprising since fungistatic drugs do not kill cells but only inhibit proliferation. Therefore, the viability of high-density, nongrowing microbial populations such as mature biofilms or stationary phase planktonic cells is expected to be unaffected by treatment with fungistatic drugs.

Echinocandins have fungicidal activity against *Candida* species [54] and we found that CAS killed 90% of exponential growing planktonic *S. cerevisiae* cells (Figure 3A) and 80% of proliferating biofilm cells (Figure 4). Despite the ability of CAS to kill exponentially growing yeast cells, CAS had no activity against mature biofilms (Figures 1 and 5). CAS inhibits 1,3-β-glucan synthase, which disrupt the yeast cell wall and result in osmotic stress and cell lysis [8]. However, the synthase is most active in growing cells [55,56], so CAS is unable to kill growth-arrested cells [54] as we observed in this study (Figure 3B) including the cells in mature biofilms.

The polyene AmB was the only drug tested in this study with activity against cells in mature biofilms (Figures 1 and 5) and growth arrested planktonic cells (Figure 3B). AmB binds to ergosterol in the cell membrane and form pores that increase the permeability of electrolytes and small molecules. Pore formation results in loss of membrane potential and eventually cell lysis [9]. Since ion diffusion and lysis are passive processes, cell metabolism is not required for AmB to kill cells. Consistent with this mechanism, AmB killed both growing and non-growing yeast cells (Figure 3). However, although AmB killed cells in non-proliferating, low-density yeast populations, AmB-tolerant subpopulations were observed in stationary state planktonic and biofilm populations (Figure 5).

Azole drugs are fungistatic against *C. albicans*, but less active against other *Candida* species, which show a clear increase in cell density after azole treatment [57,58]. The poor efficacy of the azole drug VOR against *S. cerevisiae* cells within the first 7 hours of exposure (Figure 3A) might be because *S. cerevisiae* and the closely related *C. glabrata* share a mechanism that makes them intrinsically resistant to azoles.

Bacterial cells grown as biofilms or grown to stationary state as planktonic cells have similar drug-tolerance phenotypes [40,41,59,60]; we have in the present study extended this phenotypic similarity to include yeast. Our results indicated that the biofilm mode of growth itself does not result in antifungal tolerance. Rather, the lack of cell division and the physiological state of stationary phase cells is responsible for the drug-tolerant phenotype.

## Conclusions

A combination of factors is probably responsible for the multidrug tolerance of cells in biofilms. However, as long as biofilm populations contain non-proliferating cells, some of the most commonly used antimicrobials will have reduced efficacy. Biofilm tolerance to drugs is conditional and depends on the mode of action of the tested drugs, as well as cell physiology and environment [61]. We showed that standard laboratory yeast biofilm models and methods can determine cell culture conditions under which antifungal drugs are effective or ineffective. Our findings imply that biofilm tolerance phenotypes might be caused by the large number of stationary cells within mature biofilms rather than specific biofilm mechanisms. Our data therefore suggest that future research on novel drugs and treatments should focus on strategies that are effective against stationary non-growing cells, rather than attempting to develop specific anti-biofilm treatments. The results obtained in this study are based on *in vitro* experiments and relies on the value of *S. cerevisiae* as a model organism for the pathogenic fungi. Our results indicate that *S. cerevisiae* and *C. glabrata* biofilms have similar antifungal sensitivity, but the results should also be verified in an *in vivo* model.

## Methods

### Yeast strains

*S. cerevisiae* Σ1278b YS-11 (*MAT*a *can1Δ*::*STE2p*-*spHIS5 lyp1Δ*::*STE3p*-*LEU2 his3*::*HisG leu2Δ ura3Δ*) was used as reference strain (a gift from the Boone Laboratory, University of Toronto). A *flo11* mutant that does not form biofilm was obtained from the Σ1278b gene deletion library [27]. *C. glabrata* (ATCC 90030) was obtained from the American Type Culture Collection. A strain expressing green fluorescent protein (GFP) was constructed by expressing the *GFP* gene from the *TEF1* promoter. The *TEF1* promoter was PCR amplified from pSP-GM2 [62] with primers TEF-F: 5’-CGTGCGAUGCCGCACACACCATAGCTTC and TEF-R: 5’-ACGTATCGCUGTGAGTCGTATTACGGATCCTTG. *GFP* was amplified from pJBA27a [63] with primers GFP-F: 5’-AGCGATACGUAGCATGCGTAAAGGAGAAGAA and GFP-R: 5’-CACGCGAUTATTTGTATAGTTCATCCATGCC. The *GFP* and *TEF1* DNA fragments were simultaneously fused and cloned into a digested vector with USER (uracil-specific excision reagent) technology as previously described [64,65]. In short, the vector pXI-2 [66] was digested with *Asi*SI and nicking enzyme Nb.BsmI. Ten μl of digested vector was mixed with 5 μl of each DNA fragment, 1 μl USER enzyme and 1.5 μl mili-Q water. The mix was incubated for 25 minutes at 35°C followed by 25 minutes at 25°C. Subsequently, the reaction mixture was used directly to transform competent *Escherichia coli* cells (DHα5). The resulting plasmid was denoted pRKB5. The *TEF1p*-*GFP* fragment was inserted in chromosome XI position (91,575..92,744) of the reference strain using a high-efficiency transformation protocol [67] and transformants selected on synthetic complete agar medium that did not contain uracil.

### Media and antifungals

All experiments were performed in synthetic complete medium (0.67% yeast nitrogen base supplemented with glucose and amino acids) [68], which is the standard medium for the study of *S. cerevisiae* biofilms [28]. A 0.2% (w/v) glucose concentration was used in all biofilm experiments. Yeast extract peptone dextrose (YPD) [68] agar plates were used for colony counting. Antifungals VOR, 5FC, AmB and CAS were from Sigma-Aldrich. All antifungals were dissolved in DMSO in 5 mg/ml stock solutions and stored at -20°C. All experiments were performed in triplicate.

### Minimal inhibitory concentration

Minimal inhibitory concentrations (MIC) were determined as previously described [69] with modifications. In short, two-fold dilution series of antifungal drugs were prepared in fresh synthetic complete medium with 2% glucose (w/v) and distributed into 96-well microtiter plate. Synthetic RPMI medium is recommended for MIC assays by EUCAST, but *S. cerevisiae* grows poorly in RPMI and MIC was therefore determined in synthetic complete medium. Visibly turbid overnight cultures were diluted to OD_600_ 0.1 in fresh medium and transferred to microtiter plates containing aliquots of serially diluted antifungal drug. Plates were statically incubated at 30°C for 24 h and absorbance was measured with a microplate spectrophotometer (BioTek PowerWave 340). Growth inhibition of ≥ 50% was determined as MIC for CAS, VOR and 5FC and ≥ 90% growth inhibition was determined as MIC for AmB as recommended by EUCAST [69].

### Visualization of biofilm drug susceptibility

Visibly turbid cultures were diluted to OD_600_ 0.1. After 2 hours at 30°C, cells were transferred to biofilm chambers (Technical University of Denmark) with a polyvinyl chloride (PVC) coverslip surface (Rinzl, Electron Microscopy Sciences). After 4 or 48 hours static incubation at 30°C, medium was removed from biofilm chambers and centrifuged and antifungal drug was added to the supernatants at 10 times the MIC. Spent medium with drug was introduced to biofilm cultures followed by 24 hours at 30°C. Chromosomally integrated GFP and 3 μM Syto9 (Invitrogen) were used to visualize live cells and 1 μM propidium iodide (Sigma-Aldrich) was used to stain dead cells. CLSM was performed with a Zeiss LSM710 microscope equipped with excitation lasers at 488 nm and 514 nm. Imaging used an EC Plan-Neofluar 40x/1.30 Oil lens.

### Metabolic activity

Preparation of cell cultures and CLSM imaging was as described above except a Plan-Apochromat 63x/1.40 Oil DIC M27 objective was used. Metabolically active cells were distinguished from inactive cells with 10 μM FUN-1 as described by the manufacturer (Molecular Probes, Probes for yeast viability, MP 07009). Cells were considered metabolic active if they produced red cylindrical intravacuolar structures [42,51].

### Killing kinetics

Overnight cultures were diluted to OD_600_ 0.01 in fresh synthetic medium. Yeast cultures were grown to exponential phase in baffled shake flasks at 30°C and samples were distributed to test tubes for exposure to antifungal drugs at 5 times the MIC before incubation at 30°C with aeration. Samples were extracted at indicated time-points. CFUs were determined by plating serial dilutions on YPD agar.

### Antifungal survival assay

Visibly turbid cultures were diluted to OD_600_ 0.1 in synthetic medium and grown in baffled shake flasks for 2 hours. Culture samples were distributed to glass tubes for planktonic cells and polystyrene microtiter plates for biofilms and incubated statically at 30°C. After 4 or 48 hours, cells were challenged with antifungal drug at 10 times the MIC, added in spent medium, for 24 hours. Viable cells were determined by counting CFUs on YPD agar. Biofilm cells were washed twice in saline and CFU was determined.

### Statistical analysis

Unpaired Student’s *t*-test was used for statistical analysis. P < 0.01 was considered significant. All statistical calculations were performed using GraphPad Prism version 5.00 for Windows, GraphPad Software, San Diego California USA.

## Abbreviations

VOR: Voriconazole
5FC: Flucytosine
CAS: Caspofungin
AmB: Amphotericin B
ECM: Extracellular matrix
MIC: Minimal inhibitory concentration
PI: Propidium iodide
CLSM: Confocal laser scanning microscopy
CFU: Colony forming unit
